# Ferroportin at the Crossroads of Iron Biology: Disease, Regulation and Modulation

**DOI:** 10.3390/biom16071066

**Published:** 2026-07-21

**Authors:** Pramudi Hasanga Rathnayake, Nina E. Ryan, Ryan Atkins, Daniel F. Wallace, V. Nathan Subramaniam

**Affiliations:** 1 Hepatogenomics Research Group, Queensland University of Technology (QUT), 60 Musk Ave, Kelvin Grove, Brisbane, QLD 4059, Australia; 2 Centre for Genomics and Personalised Health, School of Biomedical Sciences, Queensland University of Technology (QUT), 60 Musk Ave, Kelvin Grove, Brisbane, QLD 4059, Australia; 3 Metallogenomics Laboratory, Queensland University of Technology (QUT), 60 Musk Ave, Kelvin Grove, Brisbane, QLD 4059, Australia

**Keywords:** ferroportin, iron metabolism, iron regulation, non-HFE, haemochromatosis

## Abstract

Iron is an essential element for almost all living beings. Ferroportin is the only known cellular iron exporter and is responsible for maintaining iron homeostasis. The hepcidin-ferroportin axis is central to iron regulation. Dysregulation of ferroportin is thus associated with iron disorders. Understanding how ferroportin is regulated will provide greater insight into iron metabolism and potential therapies for iron-related disease. This review synthesizes current knowledge on ferroportin biology with a particular focus on its regulatory modulators and their therapeutic potential and provides an updated perspective on the molecular pathogenesis and clinical spectrum of ferroportin disease. Elucidating these mechanisms will be essential for the development of targeted interventions to correct iron dysregulation in diverse human diseases.

## 1. Introduction

### 1.1. Iron Metabolism

Iron is one of the most important metals for sustaining all forms of life, from single cells to multi-cellular organisms including humans [1]. It is a transition metal which has the ability to donate and accept electrons in redox reactions, making it a favourable metal for biological and cellular processes such as DNA synthesis and nucleic acid repair, and has a fundamental role in oxygen transport as a component of haemoglobin [1,2].

**Figure 1:**
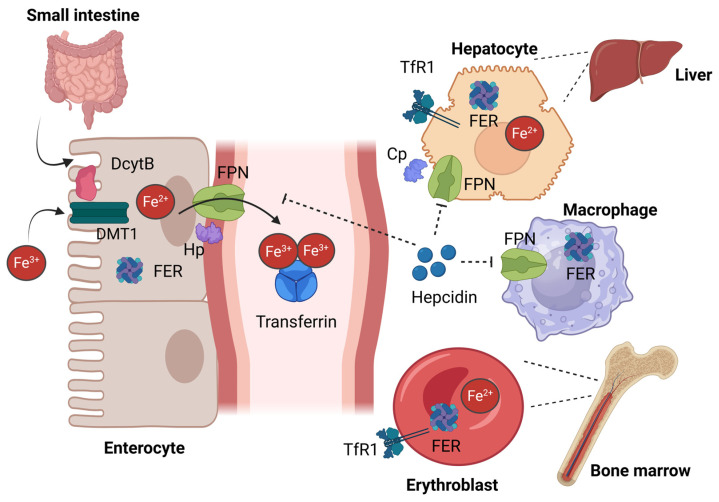
Iron regulation in the human body. Dietary iron (Fe^3+^) is reduced to Fe^2+^ by the iron reducing duodenal cytochrome B reductase (DcytB) and taken up at the apical membrane of enterocytes via divalent metal transporter (DMT1). Ferroportin (FPN) is involved in transporting iron into the circulation, which is re-oxidized by the ferroxidases such as hephaestin (Hp) or ceruloplasmin (Cp). Oxidized iron binds with transferrin (Tf) and is transported to tissues including bone marrow and liver. Erythrocytes and hepatocytes take up iron through transferrin receptor 1 (TfR1) and excess iron is stored as ferritin (FER). Senescent erythrocytes are phagocytosed and degraded by macrophages and contribute to iron recycling. Hepcidin, the main iron regulator, is produced and released by hepatocytes. Hepcidin binds to FPN and initiates its internalisation and degradation in enterocytes, macrophages and hepatocytes to regulate iron levels in the circulation. Created in BioRender.com.

**Figure 2:**
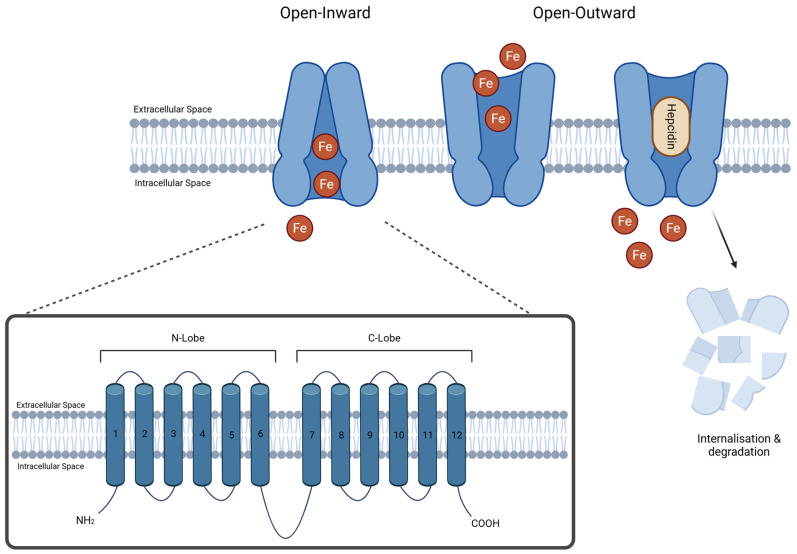
FPN transmembrane structure and conformational mechanism. FPN consists of 12 transmembrane helices bundled into 2 lobes (N- and C- lobes). The structure can switch between an open-inward conformation and open-outward conformation, which underlies its iron export mechanism. Hepcidin can only bind to the open-outward conformation, causing its internalization and degradation within the cell. Created in BioRender.com.

Iron metabolism is tightly regulated in the body to maintain an optimal iron supply for metabolic reactions. The regulation is maintained at the level of iron uptake, transport, storage and utilization, by key regulatory molecules and pathways. Key players in iron metabolism include divalent metal transporter (DMT1), ferroportin (FPN), transferrin (TF), transferrin receptor 1 (TFR1), transferrin receptor 2 (TFR2), hemojuvelin (HJV), ferritin and hepcidin (Figure 1) [1]. Iron is absorbed through the duodenum and proximal jejunum, with iron levels being balanced by regulating iron absorption [3,4]. Iron in the diet is reduced from ferric (Fe^3+^) to ferrous (Fe^2+^) iron via the membrane bound ferric reductase duodenal cytochrome B (DCYTB) and transported across the apical membrane of enterocytes by the divalent metal transporter 1 (DMT1) [4]. Ferrous iron is then transported to the circulation through the basolateral membrane of enterocytes. The only known iron export protein FPN and the membrane-bound ferroxidase hephaestin (Hp) are located at the basolateral membrane. FPN exports Fe^2+^ into the blood and Hp oxidises it to Fe^3+^, which is then bound to the iron transport protein TF. Most of the iron present in the circulation is bound to TF, and it is in this form that it enters erythrocyte precursors in the bone marrow via TFR1 and receptor-mediated endocytosis, for the primary purpose of haemoglobin synthesis. Most of the remaining circulatory iron enters the liver and is stored as ferritin [5]. As mentioned earlier, iron is an essential component of haemoglobin, and senescent red blood cells can be recycled to produce iron via phagocytosis within macrophages [4]. Ferritin-bound iron is the major form of iron storage in macrophages and hepatocytes, and both cell types are able to transport iron back into the circulation via FPN. Several other cell types including enterocytes, placental syncytiotrophoblasts, and some neurons also contribute to iron export into the circulation. In addition, liver hepatocytes act as the central regulators of iron homeostasis by producing and releasing the iron regulatory hormone hepcidin, which controls the action of FPN [6]. Hepcidin inhibits iron release from cells by binding to FPN, causing its internalization and degradation within the cell (Figure 2) [7,8]. This hepcidin-FPN axis is essential for iron homeostasis, as iron export via FPN determines the levels of iron in the circulation and is therefore predominantly responsible for the regulation of systemic iron levels [7,8].

### 1.2. Clinical Significance

Iron is essential for day-to-day physiological needs, however, if not tightly regulated, it can also be detrimental, leading to iron deficiency or overload. An excess or deficiency of iron is associated with number of clinical conditions. The human body lacks specific physiological mechanisms for iron excretion, and the levels are thus tightly regulated at the site of absorption to maintain a continuous supply of iron to the tissues. Iron related diseases are a global health and economic burden and the World Health Organization (WHO) estimates that for every US$1 invested in reducing anaemia in women, there is a potential economic return of US$12 [9].

#### 1.2.1. Iron Deficiency Anaemia (IDA)

The prevalence of anaemia in the global population is approximately 25% [10]. Iron deficiency is the most common cause of anaemia worldwide which is associated with cognitive impairment in children, reduced work productivity in adults and increased risk of maternal and neonatal complications. According to the WHO, anaemia affects about 40% of children under 5 years, 37% of pregnant women, and 30% of women of reproductive age globally. IDA accounts for nearly 50% of all anaemia cases [9]. Dietary iron deficiency alone accounts for about 16.7% percent of cases worldwide, highlighting the nutritional importance of iron [11]. These figures highlight the critical need for targeted interventions to address iron deficiency anaemia worldwide.

#### 1.2.2. Iron Overload

Iron overload disorders also present a significant clinical burden. Excessive iron deposition can lead to oxidative damage and organ dysfunction. Primary iron overload is caused by genetic defects in genes involved in iron regulation. The most common example is hereditary haemochromatosis (HH), which will be discussed later. In contrast, secondary iron overload may result from repeated blood transfusions in chronic haemolytic anaemia conditions such as thalassemia major, sickle cell disease etc, chronic liver diseases or excessive iron supplementation [1]. The primary mechanism of organ damage is increased oxidative stress due to iron-induced formation of reactive oxygen species (ROS) [12].

### 1.3. Current Treatment Options and Limitations

Current therapeutic strategies for iron disorders focus on adjusting systemic iron imbalance through iron supplementation or removal of excess iron indirectly from the body. Yet, these methods are associated with certain limitations. Iron deficiency anaemia (IDA) is commonly managed with oral iron supplementation, which is considered the first-line treatment due to its efficacy and low cost [13]. However, gastrointestinal side effects such as nausea, constipation, and abdominal discomfort often limit its effectiveness [14,15]. Intravenous iron therapy is used for patients with oral iron intolerance, malabsorption or severe anaemia [16]. This method is more expensive and requires administration under medical surveillance [17]. Further, intravenous therapy carries a risk of hypersensitivity reactions [1,18].

Treatments for iron overload include phlebotomy or iron chelation therapies [1]. Although therapeutic phlebotomy is widely used for reducing excess iron, it is time-consuming and has side effects requiring long-term maintenance [19]. Further, phlebotomy is unsuitable for patients with anaemia [20]. Additionally, iron chelation therapy, includes agents such as deferoxamine, deferasirox and deferiprone [21]. Deferoxamine is administered via intravenous or subcutaneous routes and requires prolonged infusions which limits patient compliance [22]. Deferasirox and deferiprone are provided as oral drugs. However, with prolonged administration, these therapies can lead to the development of gastrointestinal disturbances, renal and hepatic toxicity and haematological adverse effects such as agranulocytosis [23]. Furthermore, chelation removes iron slowly and inefficiently as a small fraction of total body iron is readily accessible to the drug and established organ damage is only partially reversible [24]. These limitations highlight that the currently available therapies are focused on reducing body iron levels rather than addressing the underlying condition leading to iron overload and emphasise the need for targeted therapies that address the underlying molecular mechanisms of iron homeostasis.

Scientific research continues to emphasise the importance of maintaining iron homeostasis and to explore mechanisms that could serve as therapeutic targets for conditions such as iron deficiency or overload. One key pathway is the hepcidin–FPN axis. In particular, the iron exporter protein FPN will be highlighted here for its potential as a novel therapeutic target.

## 2. Ferroportin: Structure and Mechanism

FPN is the only member of the solute carrier 40 family of membrane transporter proteins. The gene, *SLC40A1*, is located on human chromosome 2 and is translated into a 571 amino acid protein with a predicted molecular weight of 65–70 kDa [25,26,27]. The iron exporter protein consists of 12 transmembrane helices arranged into two 6 transmembrane-helix bundles, termed the N-lobe and C-lobe, connected by a cytoplasmic loop (Figure 2) [28,29,30,31]. These helix bundles are organised in the membrane to form an internal cavity, through which iron can be transported. Conformational changes to the structure of FPN underpin the mechanism of iron transport. In an inward-open conformation, the cavity is open towards the intracellular space of the cell, having the capacity to bind iron and be readily available to export when necessary [30,32]. This conformation is then altered to an outward-open model, where the cavity is open to the extracellular space, releasing the bound iron [30,32]. This fluctuation of FPN conformation means that selective binding of hepcidin to the outward-open model, resulting in FPN internalisation and degradation, only occurs based upon intracellular iron abundance [7,33]. Therefore, the active release of iron into the extracellular space can be maintained, sparing FPN molecules with lower transport activity [7].

More is known now about the mechanics of how the lobes interact with iron and hepcidin. Initial 3D models of FPN were based upon a comparison to membrane transport proteins from other species such as *E. coli* glycerol-3-phosphate transporter, GlpT [33], and the *Bdellovibrio bacteriovorus* Bd2019 iron transporter [32]. More recent studies have determined cryogenic electron microscopy (cryo-EM) structures of human FPN in complex with hepcidin and the inhibitor vamifeport and highlight the iron exporter’s conformational structures and the potential for therapeutic targets [7,34]. It is predicted that each conformation has a cluster of residues lying within the cavity to assist with FPN protein function, whether it allows for the trafficking of iron (inward-open model) and the residues involved in hepcidin binding (outward-open model) [30]. FPN has also been structurally analysed to identify two divalent metal-binding sites (one per lobe) facing the internal cavity, with cryoEM structures indicating the N-lobe sites primarily serve for iron efflux, and the C-lobe sites serve primarily for hepcidin binding [7]. The mediation of iron through these sites is still not entirely understood [7].

## 3. Hereditary Haemochromatosis

Hereditary haemochromatosis (HH) is a group of genetically inherited disorders affecting iron metabolism, caused by mutations in genes encoding proteins involved in the regulation of iron homeostasis [35]. HH has 5 subtypes based on the genes affected: HFE-related, HJV-related, HAMP-related, TFR2-related, and SLC40A1-related, with the HFE-related form being the most common in patients [35]. These subtypes were previously referred to as types 1, 2A, 2B, 3 and 4, however, the new gene-centred classification system better reflects the molecular basis of disease [36]. The HFE-, HJV-, HAMP- and TFR2-related forms of HH are autosomal recessive disorders, with the underlying genetic defects resulting in relative hepcidin deficiency, leading to enhanced iron absorption and systemic iron overload [36]. They differ in severity and age of onset, with HJV- and HAMP-related HH also referred to as juvenile haemochromatosis [37]. SLC40A1-related iron overload has some unique features compared to the other forms of HH and two distinct phenotypic subtypes which are discussed in more detail in the next section.

### SLC40A1 (FPN)-Related Iron Overload

FPN-related iron overload, previously referred to as type 4 HH is an autosomal dominant condition due to mutations in the *SLC40A1* gene. As stated earlier, there are two distinct phenotypic subtypes that are caused by either gain of function (GoF) or loss of function (LoF) mutations [33,38]. The two subtypes are distinguishable by the pattern of iron accumulation, either predominantly in macrophages (LoF) or hepatocytes (GoF). Both subtypes have elevated serum ferritin concentrations, however, patients with LoF mutations typically have a transferrin saturation (TSAT) in the low to normal range, in contrast to those with GoF mutations, where transferrin saturation is typically in the elevated range, similar to other forms of HH. GoF variants result in a FPN protein that is resistant to hepcidin [36,39,40,41]. Most GoF mutations occur close to the hepcidin binding site [29] and either prevent hepcidin binding or prevent hepcidin-induced internalisation and degradation of the protein. This means that GoF FPN protein remains unregulated even when hepcidin levels are high, resulting in continued cell surface expression and uncontrolled iron export into the bloodstream [42]. This excess iron is primarily accumulated within hepatocytes [29,42]. The iron overload phenotype of patients with GoF FPN mutations is similar to the types of HH mentioned earlier that primarily result from hepcidin insufficiency. Hence, GoF FPN-related iron overload is classified as a type of HH (FPN-related HH) because it shares the same pathophysiological basis as other types of HH [39], all being characterised by an impairment in hepcidin function, either resulting from defects to pathways upstream of hepcidin, within hepcidin itself or in the downstream target of hepcidin, FPN. The first cases of FPN-related HH were reported in a large Dutch pedigree with autosomal dominant iron overload who carried the p.N144H mutation [43], although an earlier report in a large Solomon Islands pedigree is most likely due to a mutation affecting the same amino acid (p.N144T) [44,45]. Other mutations that lead to the FPN-related HH phenotype include p.Y64N, p.C326Y, p.Y333H and p.H507R [29]. Many GoF variants decrease hepcidin binding to FPN by either affecting residues that are located in the hepcidin binding site or in the case of p.Y64N affecting the ubiquitination of FPN following hepcidin binding [7].

LoF FPN mutations affect the iron-export capabilities of FPN, either by disrupting the mechanism of iron transport or by affecting the expression or localisation of FPN on the cell surface [36,39]. With diminished iron export, iron accumulates within FPN expressing cells, such as macrophages, and there is a reduced ability to mobilise iron from stores. This helps to explain the low-to-normal TSAT levels observed in patients with LoF mutations and a tendency to develop anaemia following phlebotomy treatment [29,39].

Although, GoF and LoF mutations both lead to iron overload, the pattern and pathophysiological basis of iron loading is different, hence iron overload due to LoF FPN mutations is not considered to be a form of HH and is commonly referred to as FPN disease, being exclusively related to defects in FPN function and unrelated to hepcidin regulation or function [36]. The first FPN disease cases were reported in a large Italian pedigree with autosomal dominant iron overload in 1999 [46], which were later genetically confirmed to be caused by the p.A77D mutation [47]. The p.V162del mutation is the most commonly reported FPN disease mutation [29], other mutations that have been exclusively associated with the LoF FPN disease phenotype include p.D157N, p.W158C, p.D181N, p.S209L, p.G468S and p.R489K [29]. Many of the LoF variants affect residues within the intracellular gate of FPN, subsequently altering the conformational steps to iron efflux, however, misfolding and defective trafficking of the mutant FPN protein have also been proposed as mechanisms leading to LoF [48].

Pathogenic variants within the *SLC40A1* gene which cause FPN-related iron overload have been identified across numerous populations worldwide [39,49]. Interestingly, despite FPN disease being considered rare, current literature suggests that there is a higher prevalence than initially suspected, and it is the most common genetic iron overload syndrome after *HFE*-HH [41,49].

## 4. Modulators and Regulators of FPN

FPN activity is tightly regulated at multiple levels. While hepcidin is the main systemic regulator of FPN, a complex network of additional modulators influences its expression, localization, stability and function. These include transcriptional and post-transcriptional regulators, inflammatory and oxidative stress pathways, intracellular iron-sensing mechanisms and protein–protein interactions that modulate FPN activity in the cell.

### 4.1. Post-Translational and Post-Transcriptional Regulation of FPN

#### 4.1.1. Hepcidin

Hepcidin is the primary regulator of FPN at post-translational level and plays a central role in maintaining systemic iron homeostasis. Hepcidin is a small peptide hormone predominantly synthesised and secreted from the liver in response to circulating iron levels, inflammation and cytokines [50]. Hepcidin directly binds to the extracellular hepcidin-binding domain of FPN [51]. This binding induces a conformational change in FPN which facilitates its ubiquitination, endocytosis and subsequent lysosomal degradation [52,53]. As a result, the number of functional FPN molecules on the cell surface is reduced and thus effectively blocks iron efflux from the cells into the plasma. Thus, high hepcidin levels lead to decreased intestinal iron absorption and sequestration of iron within macrophages and hepatocytes, resulting in reduced serum iron availability. During iron deficiency, hypoxia or increased erythropoietic demand, FPN remains stabilized on the cell membrane, promoting iron export into the circulation and increasing serum iron levels [54].

FPN is further regulated at the post-translational level by the ferroxidases ceruloplasmin and hephaestin, which stabilize FPN at the cell surface while facilitating the oxidation of Fe^2+^ to Fe^3+^. In addition, emerging evidence suggests that the amyloid precursor protein (APP) may contribute to FPN stabilization and iron export [55].

#### 4.1.2. IRPs and Intracellular Iron

FPN is also regulated at the post-transcriptional level through the iron regulatory protein/iron-responsive element (IRP/IRE) system, which directly links with intracellular iron availability. The mRNA that encodes FPN contains an IRE at its 5′ untranslated region, which is sensitive to cellular iron status [56]. Under conditions of low intracellular iron, the iron regulatory proteins (IRP1 and IRP2) are activated and bind to the IRE of FPN mRNA [57]. This binding inhibits translation of FPN ensuring the suppression of iron export when intracellular iron is scarce, thereby conserving iron for essential cellular processes such as mitochondrial respiration, DNA synthesis, and enzymatic reactions [58]. Conversely, when intracellular iron levels are sufficient or high, iron directly or indirectly inhibits IRP binding activity. IRP1 incorporates an iron-sulfur cluster and functions as a cytosolic aconitase, losing its RNA-binding ability, while IRP2 undergoes iron-dependent degradation [59]. As a result, IRPs dissociate from the IRE motif, allowing increased translation of FPN mRNA, which enhances FPN protein at the cell surface [58]. This promotes iron efflux into the plasma, reducing intracellular iron overload and restoring homeostasis. Thus, the IRP/IRE system provides a rapid, reversible, and iron-sensitive regulatory mechanism that complements systemic regulation by hepcidin and transcriptional control pathways, ensuring tight coordination between cellular iron demand and export capacity.

### 4.2. Transcriptional Regulation of FPN

#### 4.2.1. NRF2

Nuclear factor erythroid 2-related factor 2 (NRF2) plays a central role in cellular defence against oxidative and inflammatory stress [60]. Under normal conditions, NRF2 is sequestered in the cytosol by Kelch-like ECH-associated protein 1 (KEAP1) which is targeted for proteasomal degradation [61]. KEAP1 is inhibited by increased cellular stress responses, and this allows NRF2 stabilization and nuclear translocation, where it binds to antioxidant response elements (AREs) in the promoter regions of target genes [60,62]. Experimental evidence suggests that FPN is a transcriptional target of NRF2 leading to enhanced FPN mRNA expression [63]. The mechanism has been clearly demonstrated in macrophages, where NRF2 activation counteracts inflammatory signals such as lipopolysaccharide-induced suppression of FPN, supporting iron recycling and limiting oxidative damage [63]. NRF2-mediated FPN regulation also plays a protective role against iron-dependent oxidative cell death (ferroptosis) by reducing the labile iron pool [64].

#### 4.2.2. BACH1

The transcriptional repressor BACH1 (BTB and CNC homology 1) suppresses FPN expression through a direct transcriptional repression mechanism at the promoter level, mainly by competing with the activator NRF2 [62]. BACH1 forms heterodimers with small Maf proteins and binds to regulatory regions of the FPN gene promoter that contains an Antioxidant Response Elements/Maf Recognition motif and blocks transcription initiation, reducing FPN mRNA. Haem, derived from haemoglobin degradation directly inhibits BACH1 causing BACH1 dissociation from NRF2 allowing it to activate FPN [65,66].

#### 4.2.3. PPARγ

Peroxisome proliferator-activated receptor gamma (PPARγ) is a nuclear receptor that acts as a transcription factor controlling lipid metabolism and macrophage function [67]. It is activated by fatty acids or synthetic ligands. Activated PPARγ forms a complex with the retinoid X receptor (RXR) and binds to target genes, including FPN [68]. Although this mechanism is not clearly explained, the binding could increase FPN mRNA and protein expression, leading to enhanced iron export from macrophages. PPARγ promotes polarization of macrophages towards the anti-inflammatory M2 state, which is characterized by high FPN levels and efficient iron release [69,70].

#### 4.2.4. HIF

Hypoxia-Inducible Factor 2-alpha (HIF-2α) is an oxygen sensitive transcription factor which responds to hypoxic conditions in cells. HIF-2α regulates several iron related proteins including FPN at the transcriptional level. Under low oxygen conditions or iron deficiency, HIF-2α is stabilized and accumulates as its degradative enzymes become inactive. Stabilized HIF-2α translocates into the nucleus and forms a complex with HIF-β which binds to hypoxia-response elements (HREs) at the promoter region of FPN, thereby enhancing transcription [71,72]. This mechanism enhances iron export to support erythropoiesis and systemic oxygen homeostasis. Other than FPN, HIF-2α is known to enhance the transcription of other iron related genes such as DMT1 and DCYTB via the same mechanism [73]. Furthermore, Chiabrando et al., showed that this transcriptional regulation of FPN is evident in duodenal cells and splenic macrophages [74].

In addition to the transcription factors discussed above, metal-responsive transcription factor 1 (MTF-1) has also been identified as a regulator of FPN transcription. MTF-1 is activated by increased intracellular transition metal ions such as zinc and oxidative stress. Upon activation, MTF-1 translocates to the nucleus and binds to metal response elements (MREs) of the FPN gene (*SLC40A1*) promoter and induces ferroportin transcription [75].

#### 4.2.5. Inflammatory Cytokines

Pro-inflammatory cytokines such as interleukin-6 (IL-6), interleukin-1β (IL-1β), and tumour necrosis factor-α (TNF-α) are released during infection or inflammation and play a central role in suppressing iron export from macrophages [76]. During inflammation IL-6 activates the JAK/STAT3 pathway in hepatocytes, leading to increased production of hepcidin, which then triggers FPN degradation in target cells [77]. Furthermore, lipopolysaccharides (LPS) derived from bacterial cell walls can regulate FPN expression in macrophages through inflammatory signalling pathways [78]. On the other hand, LPS binds with Toll-like receptor 4 (TLR4) and activates the NF-κB pathway in macrophages, which results in transcriptional repression of FPN independently of hepcidin [79]. TNF-α and IL-1β further enhance FPN suppression by promoting inflammatory signalling pathways which favour iron retention within cells [79].

#### 4.2.6. Specific MicroRNAs

MicroRNAs regulate FPN mainly at the post-transcriptional level where they bind to the 3′ untranslated region (3′ UTR) of FPN mRNA. Micro-RNA molecules such as miR-485-3p, miR-147a and miR-4735-3p have been shown to directly target and repress FPN according to recent research data [80,81,82]. Similarly, members of the miR-17 cluster such as miR-20b have been shown to regulate intestinal FPN expression which has become a potential target for intestinal iron exportation [83]. A member of same family, miR-20a, reduces FPN expression in lung cancer cells, possibly to enhance iron availability for cancer cell proliferation [84].

#### 4.2.7. BMP/SMAD Pathway (Indirect Regulation of FPN)

The BMP/SMAD signalling pathway represents the central iron-sensing mechanism in hepatocytes, and it serves as the main upstream regulator of hepcidin transcription. Thus, the pathway indirectly controls systemic FPN activity. At increased hepatic and circulatory iron levels, iron-responsive ligand BMP6 expression is upregulated and binds to type I and type II BMP receptors on the hepatocyte membrane [85]. This binding mechanism is mediated by the glycosylphosphatidylinositol-anchored co-receptor, HJV. BMP6 ligand-receptor interaction triggers phosphorylation of intracellular SMAD proteins particularly, SMAD1, SMAD5 and SMAD8, which subsequently form a complex with the common mediator SMAD4 [86]. The activated SMAD complex translocates into the nucleus and binds to regulatory elements in the promoter region of the hepcidin gene (HAMP) to facilitate its transcription [77]. Hepcidin then binds to FPN and triggers its degradation, thereby reducing iron export into plasma. This negative feedback loop in which elevated systemic iron enhances BMP/SMAD signalling, indirectly suppresses FPN to balance systemic iron levels [87]. This pathway acts as the master hepatic iron sensor, integrating signals from tissue iron stores to maintain systemic iron homeostasis. Dysregulation of this axis due to mutations in BMP6, HJV, or SMAD can lead to pathological conditions like hemochromatosis.

## 5. Current and Emerging Pharmacological Agents or Prospective Therapeutic Strategies

The hepcidin-FPN axis is a promising target for developing therapeutics for iron-related disorders [88]. Several experimental therapies such as hepcidin mimetics, hepcidin and FPN inhibitors and antibodies for hepcidin and FPN are under investigation [89]. Despite its significance, therapeutic strategies directly targeting FPN have not been extensively developed.

### 5.1. Hepcidin Mimetics/Hepcidin Agonists/Mini Hepcidins

In certain disease conditions like HH and beta thalassemia, hepcidin activity is supressed, leading to excessive iron accumulation [90]. In beta thalassemia, hepcidin is suppressed secondarily despite the elevated serum iron levels, as ineffective erythropoiesis stimulates the production of erythroid regulators such as erythroferrone which supress hepatic hepcidin expression [91]. In contrast, HH is characterized by reduced hepcidin production due to genetic mutations in iron-regulatory genes. Thus, therapeutic efforts have focused on enhancing hepcidin activity in treating iron overload. Hepcidin mimetics are emerging therapeutic agents designed to mimic the biological activity of endogenous hepcidin and induce FPN internalization and degradation. These agents effectively reduce intestinal iron absorption and iron release from macrophages leading to decreased serum iron and tissue iron loading. Rusfertide (PTG-300) is a synthetic hepcidin mimetic peptide currently under clinical investigation for conditions such as polycythemia vera and iron overload [92]. It has demonstrated the ability to reduce serum iron levels and transferrin saturation in human-clinical trials [93,94].

Mini-hepcidins, like Hep9 derivatives, are small, engineered peptides that mimic the active N-terminal region of native hepcidin [95]. These derivatives are currently under preclinical and early clinical development. Collectively, these agents have significant clinical relevance, as they offer promising therapeutic options for the management of iron overload disorders such as HH, beta thalassemia and polycythaemia vera. Further, they may serve as effective alternatives to traditional treatments like phlebotomy and iron chelation therapy.

### 5.2. Hepcidin Antagonists

Hepcidin antagonists are developed to correct iron restricted anaemias characterized by pathologically elevated hepcidin, such as anaemia of inflammation and chronic kidney disease. These agents include monoclonal antibodies and hepcidin-binding molecules which neutralize hepcidin activity to prevent its interaction with FPN. Anticalins, such as PRS-080 are engineered binding-proteins designed to neutralize hepcidin and they are currently under investigation in early clinical stages [96]. Although, studies have demonstrated increases in serum iron and transferrin saturation, their clinical development has been limited by the rapid turnover and high production rate of hepcidin in the body. In addition, anti-hepcidin monoclonal antibodies, such as LY2787106, have been developed to bind and neutralise circulating hepcidin, however, clinical studies have demonstrated only transient increases in serum iron parameters [97].

### 5.3. Hepcidin Production Enhancers

TMPRSS6 (Matriptase 2) inhibitors have become one of the most advanced and precise approaches for regulating the hepcidin-FPN axis. TMPRSS6 acts as a negative regulator of hepcidin by suppressing BMP-SMAD signalling in hepatocytes [98]. Inhibition of TMPRSS6 enhances the endogenous production of hepcidin. The monoclonal anti-TMPRSS6 antibody, REGN7999, has demonstrated robust increases in hepcidin and reductions of body iron levels in healthy volunteers [99]. Furthermore, siRNA molecules (SLN124) and antisense oligonucleotides have also been developed to suppress hepatic TMPRSS6 expression [100]. These treatments are highly liver-specific and can produce long-lasting increased hepcidin levels, which hold promise for treating chronic iron overload.

### 5.4. FPN Targeted Agents

Another therapeutic strategy involves directly targeting FPN to prevent its degradation following hepcidin binding. These agents aim to restrict iron export capacity without inhibiting hepcidin signalling. One key drug under investigation is vamifeport (VIT-2763), which is a an oral small-molecule FPN inhibitor. It is currently being evaluated in Phase 2 clinical studies [101]. Agents within this class have shown efficacy in increasing serum iron and improving erythropoiesis in iron restricted states. This drug has proven to be promising in the treatment of beta thalassemia and sickle cell disease. Additionally, Witcher et al. reported that the antibody LY2928057, which target FPN, can block hepcidin binding and restrict iron efflux [102]. However, this was not developed as a clinical drug.

Overall, these emerging therapies show a shift from traditional treatment strategies that depend on iron supplementation or chelation, toward more targeted regulation of the hepcidin-FPN axis. However, there are still relatively few studies focusing on drugs that directly target FPN, highlighting the need for further research in this area. Such approaches may offer important advantages beyond hepcidin-targeted therapies for more precise control of iron homeostasis.

## 6. Conclusions

In summary, although substantial progress has been made in understanding FPN regulation, current evidence indicates that most regulatory mechanisms act at the transcriptional level. Consequently, post-translational control of FPN, particularly processes governing its trafficking, localization, stability, and internalization, remain relatively underexplored. This gap represents a significant limitation in our understanding of FPN biology and underscores the need for further investigation. Moreover, most existing therapeutic strategies primarily target hepcidin, with comparatively few studies focusing on agents that directly modulate FPN. Expanding research efforts to identify novel direct regulators and FPN-specific therapeutic approaches could offer new avenues for treating iron-related disorders and deepen our understanding of iron homeostasis beyond the classical hepcidin–FPN paradigm.

## Data Availability

No new data were created or analyzed in this study. Data sharing is not applicable to this article.

## References

[1] Roemhild K., von Maltzahn F., Weiskirchen R., Knüchel R., von Stillfried S., Lammers T. (2021). Iron metabolism: Pathophysiology and pharmacology. Trends Pharmacol. Sci..

[2] Galy B., Conrad M., Muckenthaler M. (2024). Mechanisms controlling cellular and systemic iron homeostasis. Nat. Rev. Mol. Cell Biol..

[3] Wallace D.F. (2016). The Regulation of Iron Absorption and Homeostasis. Clin. Biochem. Rev..

[4] Mleczko-Sanecka K., Silvestri L. (2021). Cell-type-specific insights into iron regulatory processes. Am. J. Hematol..

[5] Vogt A.S., Arsiwala T., Mohsen M., Vogel M., Manolova V., Bachmann M.F. (2021). On Iron Metabolism and Its Regulation. Int. J. Mol. Sci..

[6] Qiu L., Frazer D.M., Hu M., Song R., Liu X., Qin X., Ma J., Zhou J., Tan Z., Ren F. (2025). Mechanism and regulation of iron absorption throughout the life cycle. J. Adv. Res..

[7] Billesbølle C.B., Azumaya C.M., Kretsch R.C., Powers A.S., Gonen S., Schneider S., Arvedson T., Dror R.O., Cheng Y., Manglik A. (2020). Structure of hepcidin-bound ferroportin reveals iron homeostatic mechanisms. Nature.

[8] Nemeth E., Tuttle M.S., Powelson J., Vaughn M.B., Donovan A., Ward D.M., Ganz T., Kaplan J. (2004). Hepcidin regulates cellular iron efflux by binding to ferroportin and inducing its internalization. Science.

[9] World Health Organisation (2025). Anaemia. https://www.who.int/news-room/fact-sheets/detail/anaemia.

[10] Luo W., Sun H., Cao H., Zhou G., Luo Y. (2025). Global, regional, and national burden of anemia, 1990 to 2021: An observational study analysis for the global burden of disease. Medicine.

[11] Lee S., Son Y., Hwang J., Kim M.S., Il Shin J., Yon D.K., Kassebaum N.J. (2025). Global, regional and national burden of dietary iron deficiency from 1990 to 2021: A Global Burden of Disease study. Nat. Med..

[12] Kawabata T. (2022). Iron-Induced Oxidative Stress in Human Diseases. Cells.

[13] Tan X., Tian Y., Zhang T., Yao Q., Zhu T., Wang W., Wang Q., Fu H. (2026). Oral iron preparations: Gastrointestinal adverse events and medication adherence in female patients with iron deficiency anemia. Front. Pharmacol..

[14] Lo J.O., Benson A.E., Martens K.L., Hedges M.A., McMurry H.S., DeLoughery T., Aslan J.E., Shatzel J.J. (2023). The role of oral iron in the treatment of adults with iron deficiency. Eur. J. Haematol..

[15] Bloor S.R., Schutte R., Hobson A.R. (2021). Oral Iron Supplementation—Gastrointestinal Side Effects and the Impact on the Gut Microbiota. Microbiol. Res..

[16] Maas L.A., Krishna M., Parian A.M. (2023). Ironing It All Out: A Comprehensive Review of Iron Deficiency Anemia in Inflammatory Bowel Disease Patients. Dig. Dis. Sci..

[17] Taylor S., Rampton D. (2015). Treatment of iron deficiency anemia: Practical considerations. Pol. Arch. Med. Wewn..

[18] Lee E.J., Louden L., Applequist J., Wilson B.J., Strauss J. (2025). Perspectives on Intravenous Iron Therapy Logistics and Adherence: Results from a Patient Survey. Patient Relat. Outcome Meas..

[19] Kim K.H., Oh K.Y. (2016). Clinical applications of therapeutic phlebotomy. J. Blood Med..

[20] Romón I., Domíguez-García J., Fernández C., Carretón M., Martínez N., Calzada L., Cortés M.A., Mendez G.A., Gorostidi I., Briz M. (2024). Adverse effects of therapeutic phlebotomies: Prospective study of 587 procedures. Transfusion.

[21] Kwiatkowski J.L. (2023). Clinical Challenges with Iron Chelation in Beta Thalassemia. Hematol. Oncol. Clin..

[22] Shah N.R. (2017). Advances in iron chelation therapy: Transitioning to a new oral formulation. Drugs Context.

[23] Kontoghiorghes G.J. (2023). Drug Selection and Posology, Optimal Therapies and Risk/Benefit Assessment in Medicine: The Paradigm of Iron-Chelating Drugs. Int. J. Mol. Sci..

[24] Salimi Z., Afsharinasab M., Rostami M., Eshaghi Milasi Y., Mousavi Ezmareh S.F., Sakhaei F., Mohammad-Sadeghipour M., Rasooli Manesh S.M., Asemi Z. (2024). Iron chelators: As therapeutic agents in diseases. Ann. Med. Surg..

[25] Abboud S., Haile D.J. (2000). A novel mammalian iron-regulated protein involved in intracellular iron metabolism. J. Biol. Chem..

[26] Donovan A., Brownlie A., Zhou Y., Shepard J., Pratt S.J., Moynihan J., Paw B.H., Drejer A., Barut B., Zapata A. (2000). Positional cloning of zebrafish ferroportin1 identifies a conserved vertebrate iron exporter. Nature.

[27] McKie A.T., Marciani P., Rolfs A., Brennan K., Wehr K., Barrow D., Miret S., Bomford A., Peters T.J., Farzaneh F. (2000). A novel duodenal iron-regulated transporter, IREG1, implicated in the basolateral transfer of iron to the circulation. Mol. Cell.

[28] Shen J., Wilbon A.S., Zhou M., Pan Y. (2023). Mechanism of Ca^2+^ transport by ferroportin. eLife.

[29] Troppmair M.R., Ricci A., Scarlini S., Pelucchi S., Porto G., Busti F., Sanchez M., Weissensteiner H., Schönherr S., Forer L. (2026). Characterization of ferroportin disease and SLC40A1-related hemochromatosis—Results from the EASL non-HFE registry. J. Hepatol..

[30] Le Tertre M., Elbahnsi A., Ged C., Uguen K., Gourlaouen I., Férec C., Ka C., Le Gac G., Callebaut I. (2025). Identification of New Key Players for Ferrous Iron Export in the Asymmetric Inner Gate of Human Ferroportin 1. FASEB J..

[31] Wallace D.F., Harris J.M., Subramaniam V.N. (2010). Functional analysis and theoretical modeling of ferroportin reveals clustering of mutations according to phenotype. Am. J. Physiol. Cell Physiol..

[32] Taniguchi R., Kato H.E., Font J., Deshpande C.N., Wada M., Ito K., Ishitani R., Jormakka M., Nureki O. (2015). Outward- and inward-facing structures of a putative bacterial transition-metal transporter with homology to ferroportin. Nat. Commun..

[33] Aschemeyer S., Qiao B., Stefanova D., Valore E.V., Sek A.C., Ruwe T.A., Vieth K.R., Jung G., Casu C., Rivella S. (2018). Structure-function analysis of ferroportin defines the binding site and an alternative mechanism of action of hepcidin. Blood.

[34] Lehmann E.F., Liziczai M., Drożdżyk K., Altermatt P., Langini C., Manolova V., Sundstrom H., Dürrenberger F., Dutzler R., Manatschal C. (2023). Structures of ferroportin in complex with its specific inhibitor vamifeport. eLife.

[35] Brissot P., Pietrangelo A., Adams P.C., de Graaff B., McLaren C.E., Loréal O. (2018). Haemochromatosis. Nat. Rev. Dis. Prim..

[36] Girelli D., Busti F., Brissot P., Cabantchik I., Muckenthaler M.U., Porto G. (2022). Hemochromatosis classification: Update and recommendations by the BIOIRON Society. Blood.

[37] Sandhu K., Flintoff K., Chatfield M.D., Dixon J.L., Ramm L.E., Ramm G.A., Powell L.W., Subramaniam V.N., Wallace D.F. (2018). Phenotypic analysis of hemochromatosis subtypes reveals variations in severity of iron overload and clinical disease. Blood.

[38] Schimanski L.M., Drakesmith H., Merryweather-Clarke A.T., Viprakasit V., Edwards J.P., Sweetland E., Bastin J.M., Cowley D., Chinthammitr Y., Robson K.J. (2005). In vitro functional analysis of human ferroportin (FPN) and hemochromatosis-associated FPN mutations. Blood.

[39] Pietrangelo A. (2017). Ferroportin disease: Pathogenesis, diagnosis and treatment. Haematologica.

[40] Landemaine A., Hamdi-Roze H., Cunat S., Loustaud-Ratti V., Causse X., Si Ahmed S.N., Drénou B., Bureau C., Pelletier G., De Kerguenec C. (2022). A simple clinical score to promote and enhance ferroportin disease screening. J. Hepatol..

[41] Mayr R., Janecke A.R., Schranz M., Griffiths W.J., Vogel W., Pietrangelo A., Zoller H. (2010). Ferroportin disease: A systematic meta-analysis of clinical and molecular findings. J. Hepatol..

[42] Uguen K., Le Tertre M., Tchernitchko D., Elbahnsi A., Maestri S., Gourlaouen I., Férec C., Ka C., Callebaut I., Le Gac G. (2024). The dual loss and gain of function of the FPN1 iron exporter results in the ferroportin disease phenotype. Hum. Genet. Genom. Adv..

[43] Njajou O.T., Vaessen N., Joosse M., Berghuis B., van Dongen J.W., Breuning M.H., Snijders P.J., Rutten W.P., Sandkuijl L.A., Oostra B.A. (2001). A mutation in SLC11A3 is associated with autosomal dominant hemochromatosis. Nat. Genet..

[44] Eason R.J., Adams P.C., Aston C.E., Searle J. (1990). Familial iron overload with possible autosomal dominant inheritance. Aust. N. Z. J. Med..

[45] Arden K.E., Wallace D.F., Dixon J.L., Summerville L., Searle J.W., Anderson G.J., Ramm G.A., Powell L.W., Subramaniam V.N. (2003). A novel mutation in ferroportin1 is associated with haemochromatosis in a Solomon Islands patient. Gut.

[46] Pietrangelo A., Montosi G., Totaro A., Garuti C., Conte D., Cassanelli S., Fraquelli M., Sardini C., Vasta F., Gasparini P. (1999). Hereditary hemochromatosis in adults without pathogenic mutations in the hemochromatosis gene. N. Engl. J. Med..

[47] Montosi G., Donovan A., Totaro A., Garuti C., Pignatti E., Cassanelli S., Trenor C.C., Gasparini P., Andrews N.C., Pietrangelo A. (2001). Autosomal-dominant hemochromatosis is associated with a mutation in the ferroportin (SLC11A3) gene. J. Clin. Investig..

[48] Guellec J., Elbahnsi A., Tertre M.L., Uguen K., Gourlaouen I., Férec C., Ka C., Callebaut I., Gac G.L. (2019). Molecular model of the ferroportin intracellular gate and implications for the human iron transport cycle and hemochromatosis type 4A. FASEB. J..

[49] Wallace D.F., Subramaniam V.N. (2016). The global prevalence of HFE and non-HFE hemochromatosis estimated from analysis of next-generation sequencing data. Genet. Med..

[50] Sangkhae V., Nemeth E. (2017). Regulation of the Iron Homeostatic Hormone Hepcidin. Adv. Nutr..

[51] Zhang D.L., Senecal T., Ghosh M.C., Ollivierre-Wilson H., Tu T., Rouault T.A. (2011). Hepcidin regulates ferroportin expression and intracellular iron homeostasis of erythroblasts. Blood.

[52] Qiao B., Sugianto P., Fung E., Del-Castillo-Rueda A., Moran-Jimenez M.J., Ganz T., Nemeth E. (2012). Hepcidin-induced endocytosis of ferroportin is dependent on ferroportin ubiquitination. Cell Metab..

[53] Ross S.L., Tran L., Winters A., Lee K.J., Plewa C., Foltz I., King C., Miranda L.P., Allen J., Beckman H. (2012). Molecular mechanism of hepcidin-mediated ferroportin internalization requires ferroportin lysines, not tyrosines or JAK-STAT. Cell Metab..

[54] Xu Y., Alfaro-Magallanes V.M., Babitt J.L. (2021). Physiological and pathophysiological mechanisms of hepcidin regulation: Clinical implications for iron disorders. Br. J. Haematol..

[55] McCarthy R.C., Park Y.H., Kosman D.J. (2014). sAPP modulates iron efflux from brain microvascular endothelial cells by stabilizing the ferrous iron exporter ferroportin. EMBO Rep..

[56] Liu X.B., Hill P., Haile D.J. (2002). Role of the ferroportin iron-responsive element in iron and nitric oxide dependent gene regulation. Blood Cells Mol. Dis..

[57] Wang L., Liu X., You L.H., Ci Y.Z., Chang S., Yu P., Gao G., Chang Y.Z. (2019). Hepcidin and iron regulatory proteins coordinately regulate ferroportin 1 expression in the brain of mice. J. Cell Physiol..

[58] Maio N., Zhang D.L., Ghosh M.C., Jain A., SantaMaria A.M., Rouault T.A. (2021). Mechanisms of cellular iron sensing, regulation of erythropoiesis and mitochondrial iron utilization. Semin. Hematol..

[59] Wang J., Fillebeen C., Chen G., Biederbick A., Lill R., Pantopoulos K. (2007). Iron-dependent degradation of apo-IRP1 by the ubiquitin-proteasome pathway. Mol. Cell Biol..

[60] Franci L., Vallini G., Bertolino F.M., Cicaloni V., Inzalaco G., Cicogni M., Tinti L., Calabrese L., Barone V., Salvini L. (2024). MAPK15 controls cellular responses to oxidative stress by regulating NRF2 activity and expression of its downstream target genes. Redox Biol..

[61] Kerins M.J., Ooi A. (2018). The Roles of NRF2 in Modulating Cellular Iron Homeostasis. Antioxid. Redox Signal.

[62] Namgaladze D., Fuhrmann D.C., Brüne B. (2022). Interplay of Nrf2 and BACH1 in inducing ferroportin expression and enhancing resistance of human macrophages towards ferroptosis. Cell Death Discov..

[63] Harada N., Kanayama M., Maruyama A., Yoshida A., Tazumi K., Hosoya T., Mimura J., Toki T., Maher J.M., Yamamoto M. (2011). Nrf2 regulates ferroportin 1-mediated iron efflux and counteracts lipopolysaccharide-induced ferroportin 1 mRNA suppression in macrophages. Arch. Biochem. Biophys..

[64] Jiang X., Yu M., Wang W.K., Zhu L.Y., Wang X., Jin H.C., Feng L.F. (2024). The regulation and function of Nrf2 signaling in ferroptosis-activated cancer therapy. Acta Pharmacol. Sin..

[65] Marro S., Chiabrando D., Messana E., Stolte J., Turco E., Tolosano E., Muckenthaler M.U. (2010). Heme controls ferroportin1 (FPN1) transcription involving Bach1, Nrf2 and a MARE/ARE sequence motif at position -7007 of the FPN1 promoter. Haematologica.

[66] Kasai S., Mimura J., Ozaki T., Itoh K. (2018). Emerging Regulatory Role of Nrf2 in Iron, Heme, and Hemoglobin Metabolism in Physiology and Disease. Front. Vet. Sci..

[67] Okreglicka K., Iten I., Pohlmeier L., Onder L., Feng Q., Kurrer M., Ludewig B., Nielsen P., Schneider C., Kopf M. (2021). PPARγ is essential for the development of bone marrow erythroblastic island macrophages and splenic red pulp macrophages. J. Exp. Med..

[68] Li Z., Luo L., Yu W., Li P., Ou D., Liu J., Ma H., Sun Q., Liang A., Huang C. (2022). PPARγ phase separates with RXRα at PPREs to regulate target gene expression. Cell Discov..

[69] Lefterova M.I., Steger D.J., Zhuo D., Qatanani M., Mullican S.E., Tuteja G., Manduchi E., Grant G.R., Lazar M.A. (2010). Cell-specific determinants of peroxisome proliferator-activated receptor γ function in adipocytes and macrophages. Mol. Cell Biol..

[70] Recalcati S., Locati M., Marini A., Santambrogio P., Zaninotto F., De Pizzol M., Zammataro L., Girelli D., Cairo G. (2010). Differential regulation of iron homeostasis during human macrophage polarized activation. Eur. J. Immunol..

[71] Mastrogiannaki M., Matak P., Delga S., Deschemin J.C., Vaulont S., Peyssonnaux C. (2012). Deletion of HIF-2α in the enterocytes decreases the severity of tissue iron loading in hepcidin knockout mice. Blood.

[72] Taylor M., Qu A., Anderson E.R., Matsubara T., Martin A., Gonzalez F.J., Shah Y.M. (2011). Hypoxia-inducible factor-2α mediates the adaptive increase of intestinal ferroportin during iron deficiency in mice. Gastroenterology.

[73] Shah Y.M., Matsubara T., Ito S., Yim S.H., Gonzalez F.J. (2009). Intestinal hypoxia-inducible transcription factors are essential for iron absorption following iron deficiency. Cell Metab..

[74] Chiabrando D., Fiorito V., Marro S., Silengo L., Altruda F., Tolosano E. (2013). Cell-specific regulation of Ferroportin transcription following experimentally-induced acute anemia in mice. Blood Cells Mol. Dis..

[75] Troadec M.B., Ward D.M., Lo E., Kaplan J., De Domenico I. (2010). Induction of FPN1 transcription by MTF-1 reveals a role for ferroportin in transition metal efflux. Blood.

[76] Ganz T., Nemeth E. (2015). Iron homeostasis in host defence and inflammation. Nat. Rev. Immunol..

[77] Charlebois E., Pantopoulos K. (2021). Iron overload inhibits BMP/SMAD and IL-6/STAT3 signaling to hepcidin in cultured hepatocytes. PLoS ONE.

[78] Xia Y., Li Y., Wu X., Zhang Q., Chen S., Ma X., Yu M. (2021). Ironing Out the Details: How Iron Orchestrates Macrophage Polarization. Front. Immunol..

[79] Marques O., Horvat N.K., Zechner L., Colucci S., Sparla R., Zimmermann S., Neufeldt C.J., Altamura S., Qiu R., Müdder K. (2025). Inflammation-driven NF-κB signaling represses ferroportin transcription in macrophages via HDAC1 and HDAC3. Blood.

[80] Sangokoya C., Doss J.F., Chi J.T. (2013). Iron-responsive miR-485-3p regulates cellular iron homeostasis by targeting ferroportin. PLoS Genet..

[81] Xu P., Ge F.H., Li W.X., Xu Z., Wang X.L., Shen J.L., Xu A.B., Hao R.R. (2022). MicroRNA-147a Targets SLC40A1 to Induce Ferroptosis in Human Glioblastoma. Anal. Cell Pathol..

[82] Zhu C., Song Z., Chen Z., Lin T., Lin H., Xu Z., Ai F., Zheng S. (2022). MicroRNA-4735-3p Facilitates Ferroptosis in Clear Cell Renal Cell Carcinoma by Targeting SLC40A1. Anal. Cell Pathol..

[83] Jiang S., Fang X., Liu M., Ni Y., Ma W., Zhao R. (2019). MiR-20b Down-Regulates Intestinal Ferroportin Expression In Vitro and In Vivo. Cells.

[84] Babu K.R., Muckenthaler M.U. (2016). MiR-20a regulates expression of the iron exporter ferroportin in lung cancer. J. Mol. Med..

[85] Parrow N.L., Fleming R.E. (2014). Bone morphogenetic proteins as regulators of iron metabolism. Annu. Rev. Nutr..

[86] Xiao X., Alfaro-Magallanes V.M., Babitt J.L. (2020). Bone morphogenic proteins in iron homeostasis. Bone.

[87] Silvestri L., Nai A., Dulja A., Pagani A. (2019). Hepcidin and the BMP-SMAD pathway: An unexpected liaison. Vitam. Horm..

[88] Hawula Z.J., Wallace D.F., Subramaniam V.N., Rishi G. (2019). Therapeutic Advances in Regulating the Hepcidin/Ferroportin Axis. Pharmaceuticals.

[89] Sandnes M., Reikvam H. (2024). Hepcidin as a therapeutic target in iron overload. Expert Opin. Ther. Targets.

[90] Katsarou A., Pantopoulos K. (2018). Hepcidin Therapeutics. Pharmaceuticals.

[91] Jones E., Pasricha S.R., Allen A., Evans P., Fisher C.A., Wray K., Premawardhena A., Bandara D., Perera A., Webster C. (2015). Hepcidin is suppressed by erythropoiesis in hemoglobin E β-thalassemia and β-thalassemia trait. Blood.

[92] Modi N.B., Shames R., Lickliter J.D., Gupta S. (2024). Pharmacokinetics, pharmacodynamics, and tolerability of an aqueous formulation of rusfertide (PTG-300), a hepcidin mimetic, in healthy volunteers: A double-blind first-in-human study. Eur. J. Haematol..

[93] Kremyanskaya M., Kuykendall A.T., Pemmaraju N., Ritchie E.K., Gotlib J., Gerds A., Palmer J., Pettit K., Nath U.K., Yacoub A. (2024). Rusfertide, a Hepcidin Mimetic, for Control of Erythrocytosis in Polycythemia Vera. N. Engl. J. Med..

[94] Modi N.B., Dinh P., Xue H., Darpo B. (2025). Evaluation of Rusfertide, a Hepcidin Mimetic, on Cardiac Repolarization: A Randomized, Placebo- and Positive-Controlled Crossover Thorough QT Study in Healthy Participants. Clin. Ther..

[95] Preza G.C., Ruchala P., Pinon R., Ramos E., Qiao B., Peralta M.A., Sharma S., Waring A., Ganz T., Nemeth E. (2011). Minihepcidins are rationally designed small peptides that mimic hepcidin activity in mice and may be useful for the treatment of iron overload. J. Clin. Investig..

[96] Renders L., Budde K., Rosenberger C., van Swelm R., Swinkels D., Dellanna F., Feuerer W., Wen M., Erley C., Bader B. (2019). First-in-human Phase I studies of PRS-080#22, a hepcidin antagonist, in healthy volunteers and patients with chronic kidney disease undergoing hemodialysis. PLoS ONE.

[97] Vadhan-Raj S., Abonour R., Goldman J.W., Smith D.A., Slapak C.A., Ilaria R.L., Tiu R.V., Wang X., Callies S., Cox J. (2017). A first-in-human phase 1 study of a hepcidin monoclonal antibody, LY2787106, in cancer-associated anemia. J. Hematol. Oncol..

[98] Ganz T., Nemeth E., Rivella S., Goldberg P., Dibble A.R., McCaleb M.L., Guo S., Monia B.P., Barrett T.D. (2023). TMPRSS6 as a Therapeutic Target for Disorders of Erythropoiesis and Iron Homeostasis. Adv. Ther..

[99] Lob H.E., Singh N., Mohammadi K., Ivanova L., Crowell B., Kim H.J., Kravets L., Das N.M., Ray Y., Kim J.H. (2025). A TMPRSS6-inhibiting mAb improves disease in a β-thalassemia mouse model and reduces iron in healthy humans. JCI Insight.

[100] Porter J.B., Scrimgeour A., Martinez A., James L., Aleku M., Wilson R., Muckenthaler M., Boyce M., Wilkes D., Schaeper U. (2023). SLN124, a GalNAc conjugated 19-mer siRNA targeting tmprss6, reduces plasma iron and increases hepcidin levels of healthy volunteers. Am. J. Hematol..

[101] Pilo F., Angelucci E. (2024). Vamifeport: Monography of the First Oral Ferroportin Inhibitor. J. Clin. Med..

[102] Witcher D.R., Leung D., Hill K.A., De Rosa D.C., Xu J., Manetta J., Wroblewski V.J., Benschop R.J. (2013). LY2928057, An Antibody Targeting Ferroportin, Is a Potent Inhibitor Of Hepcidin Activity and Increases Iron Mobilization In Normal Cynomolgus Monkeys. Blood.

